# Questioning the near-intrinsic thermal conductivity of suspended graphene membranes fabricated via a cyclododecane-based transfer method

**DOI:** 10.1038/s41467-026-70213-9

**Published:** 2026-03-27

**Authors:** Mohsen Moazzami Gudarzi, Seyed Hamed Aboutalebi

**Affiliations:** 1 National Graphene Institute, University of Manchester, Manchester, UK; 2 Department of Physics and Astronomy, School of Natural Sciences, The University of Manchester, Manchester, UK; 3 Condensed Matter National Laboratory, Institute for Research in Fundamental Sciences, Tehran, Iran; 4 School of Quantum Physics and Matter, Institute for Research in Fundamental Sciences, Tehran, Iran

**Keywords:** Mechanical and structural properties and devices, Surfaces, interfaces and thin films

**arising from** Z. Wang et al*. Nature Communications* 10.1038/s41467-024-51331-8 (2024)

A recent study by Wang et al.^1^ reported a remarkably high thermal conductivity of 4914 W/m K near room temperature (338 K) in suspended graphene synthesized via chemical vapor deposition (CVD). This value notably exceeds all previous measurements for isotopically pure diamond^2^ and graphene^3^ and even surpasses theoretical predictions for pristine, infinitely large graphene sheets^4^. Wang et al. attribute this exceptional thermal conductivity to an optimized, contamination-free transfer process of their CVD-grown graphene^1^. However, based on our critical analysis of their data, we contend that the evidence presented does not substantiate this claim. Our assessment suggests that the actual thermal conductivity of their sample is substantially lower than reported, raising important questions about the reliability of these findings and the conditions necessary to achieve such high thermal transport in graphene.

The authors used Raman thermometry to probe the temperature rise in a suspended graphene heated by laser. The thermal impedance is related to the ratio of the temperature rise at a given heat input. Based on the data presented in Supplementary Fig. 18 from ref. ^1^, the temperature rise, $\triangle T$, for a given incident laser power, $\triangle Q$, can be expressed as:

$$\frac{\triangle T}{\triangle Q}=\frac{-4.83\frac{{{\mathrm{cm}}}^{-1}}{{\mathrm{mW}}}}{-0.069\frac{{{\mathrm{cm}}}^{-1}}{{{\rm{K}}}}}=70\frac{{{\rm{K}}}}{{\mathrm{mW}}}$$

However, the absorbed laser power is not explicitly provided. Assuming graphene absorbs about 3% of light at 532 nm^3^, which is an upper estimate^5^, we calculate the thermal conductivity using Eq. (1) in ref. ^1^:

$$\kappa=\frac{{\mathrm{ln}}(\frac{R}{{r}_{0}})}{2\pi d\frac{\triangle T}{\triangle Q}}\alpha=\frac{{\mathrm{ln}}(\frac{5\,{\mathrm{\mu m}}}{0.17\,{\mathrm{\mu m}}})}{2\pi \times 0.335\,{\mathrm{nm}}\times 70\frac{{{\rm{K}}}}{{\mathrm{mW}}}\times \frac{1}{0.03}}0.98=675\frac{{{\rm{W}}}}{{{\rm{m}}}\cdot {{\rm{K}}}}$$

Where $R$, ${r}_{0}$ and $d$ are the radius of suspended area, the laser beam size, and graphene thickness, respectively. $\alpha$ is a numerical factor close to unity. The calculated thermal conductivity is more than seven times smaller than the reported thermal conductivity at 338 K (Fig. 1). Such a significant discrepancy cannot be attributed to uncertainties in absorbed power alone^5^, casting doubt on how the authors arrived at their reported value of 4914 W/m K. Note that accounting for any heat loss to air would result in even smaller thermal conductivity; however, this effect is minimal for graphene^6^.

**Fig. 1:**
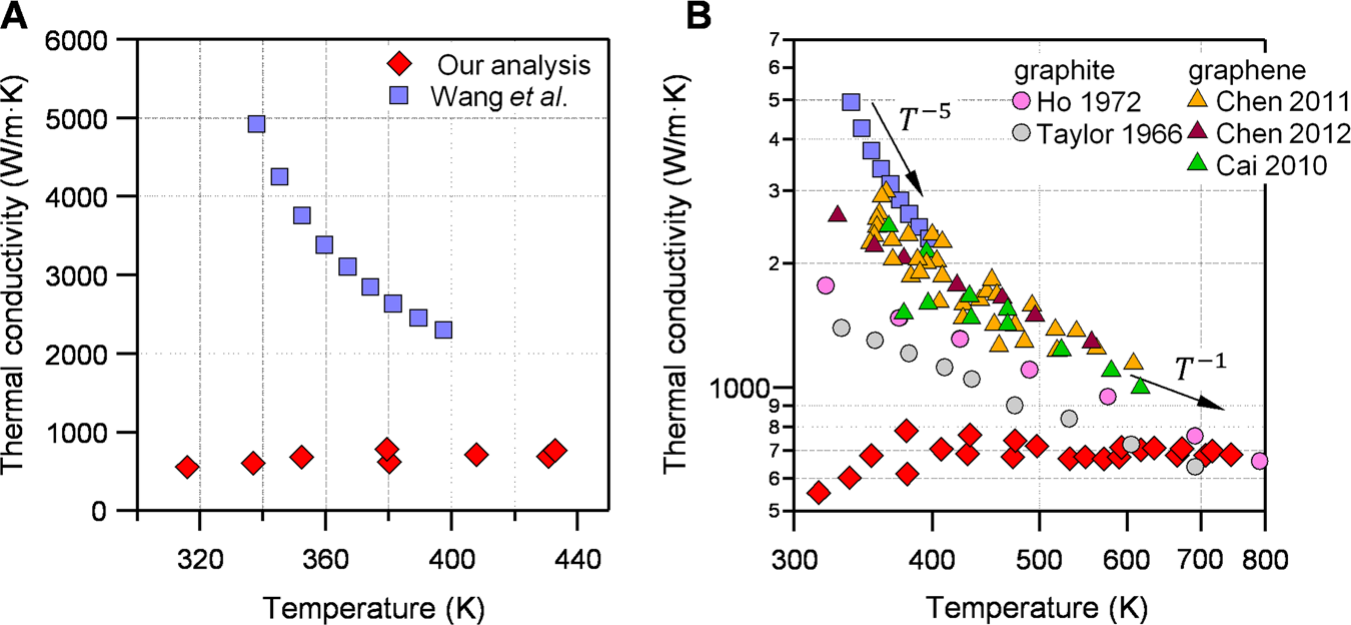
Analysis of thermal conductivity. **A** compares the thermal conductivity of graphene reported by Wang et al.^1^ and those analyzed in this work. We have plotted thermal conductivity as a function of spot temperature. We used the reported data in Supplementary Fig. 18 of ref. ^1^ to compute thermal conductivity. In **B**, the same data are presented in a logarithmic scale, in addition to literature data on the thermal conductivity of graphene and graphite. Arrows show two power-law regimes. Data for graphite are taken from refs. ^8,9^, and for graphene are taken from refs. ^3,6,10^ for natural carbon.

Furthermore, the 2D band shift shows a linear dependence on the incident laser power (Supplementary Fig. 18b from ref. ^1^), implying that the term $\frac{\triangle T}{\triangle Q}$ is constant and independent of temperature. Consequently, the thermal conductivity should also remain constant over the measured range (with a maximum temperature rise of approximately 450 K). However, Fig. 3f of ref. ^1^ presents conflicting data, indicating a more than two-fold decrease in thermal conductivity as the temperature increases from 338 K to 398 K, following a nearly T^−5^ trend (Fig. 1B). Such a drastic reduction is neither supported by the data in Supplementary Fig. 18b nor expected at temperatures near room temperature, where phonon-phonon scattering dominates^7^. Most of the previous Raman thermometry measurements on graphene showed nearly T^−1^ trend close to room temperature, similar to graphite (Fig. 1B)^3,6,8–10^. Furthermore, the inferred temperature-independent thermal conductivity in Wang et al.’s data strongly points to phonon-defect scattering as the dominant factor^7^, as the scattering rate is independent of phonon energy, casting doubt on the authors’ claims regarding the high quality and near-intrinsic thermal behavior of their suspended graphene samples.

Additionally, considering the reported sample preparation method, graphene was transferred onto a silicon nitride (SiNx) grid of unspecified thickness. Assuming a typical thickness of 100 to 200 nm for SiNx grids and acknowledging its very low thermal conductivity (~2 W/m K)^11^, it is apparent that the thermal impedance of the SiNx layer could be comparable to or even more than that of monolayer graphene (*κ* ≈ 2000 W/m K and *d* = 0.335 nm). Thus, even with ideal thermal contact between the graphene and the SiNx, the SiNx grids are unsuitable as effective heat sinks. Consequently, during laser heating, it is highly probable that the temperature of the SiNx substrate increases significantly. This calls into question the validity of applying Eq. (1) in ref. ^1^ under these experimental conditions. This is likely the reason for the observation of apparent low thermal conductivity of 675 W/m K.

In conclusion, the assumptions made regarding the thermal properties and sample preparation in this work appear to be flawed, casting substantial doubt on the accuracy of the reported thermal conductivity values.

## Data Availability

All data generated or analyzed during this study are included in the published article. All relevant processed data are available from the authors upon request.
